# Assessing the Values of Blueberries Intake on Exercise Performance, TAS, and Inflammatory Factors

**Published:** 2018-07

**Authors:** Chan Ho PARK, Yi Sub KWAK, Han Kyo SEO, Hye Young KIM

**Affiliations:** 1. Sports Science Center, Pukyong National University, Busan, Republic of Korea; 2. Dept. of Physical Education, Dong-eui University, Busan, Republic of Korea; 3. Dept. of Beauty Health Science, Shin Han University, Uijeongbu-si, Republic of Korea,; 4. Dept. of Dental Hygiene, College of Health Science, Kangwon National University, Chuncheon-si, Republic of Korea

**Keywords:** Blueberries intake, TAS, Inflammatory factors, Exercise performance, South Korea

## Abstract

**Background:**

The purpose of this study was to determine the effect of blueberry supplementation on exercise performance time, inflammation markers, energy substrates, insulin, and TAS levels during two periods: non-supplemented period and supplemented period.

**Methods:**

Eight young active participants were recruited from the Department of Physical Education at some universities in Busan City, Republic of Korea. The test period was divided into two Sections: non-supplemented period and supplemented period. Vo2 max and exercise performance time of participants were measured, with or without blueberry supplementation, with a portable gas analyzer and ECG, respectively.

**Results:**

Vo2 max and exercise performance time were increased in the blueberry supplementation period. IL-6 and CRP levels were significantly lowered in blueberry supplementation period following exercise.

**Conclusion:**

The blueberry supplementation can potentially increase the exercise performance and decrease the IL-6 and CRP levels caused by an increased TAS level.

## Introduction

Blueberry fruit is not only sweet with good flavor, but also contains dietary fiber, vitamins, protein, and nutrient materials that are good for health, including immunomodulatory agents, tannic acid, folic acid, antibacterial ingredients, and antioxidants (1). Blueberries are rich in bioactive compounds and response modifiers. Blueberry contains polyphenolic compounds (most prominently anthocyanins) with antioxidant, anti-fatigue, and inflammatory effects (2). Anthocyanins are associated with increased neuronal signaling in brain, functional mobility, mediating memory function, neurodegenerative disease, and dementia. In elderly people, limited functional mobility is associated with declines in motor and psychomotor function (3). Supplementation with polyphenolic-rich food can improve motor and psychomotor function. Preliminary studies have suggested that blueberry supplementation can improve memory and cognitive function (2) as well as brain function via decreasing oxidative stress and inflammation (4). Blueberry fruit has polyphenolics that can suppress fatigue and oxidative stress-induced muscle damage (5) as well as oxidative stress in several tissues. Consumption of blueberries can also improve blood pressure and arterial stiffness through increasing nitric oxide production (6).

The effect of blueberry intake on sports science has been determined in recent years. In animal studies, blueberry supplementation has been demonstrated to be able to improve psychomotor and motor functions (3, 7, 8). Biological activities of polysaccharides from blueberry have not been reported yet, especially in the fields of exercise performance.

It New Zealand blueberry intake can aid recovery from eccentric exercise–induced muscle damage (9). Blueberry intake could accelerate the recovery of muscle peak isometric strength. A polyphenol-enriched protein power could improve exercise-induced inflammation and oxidative stress during heavy exercise exertion in athletes. In addition, diet containing blueberriesimpacts the cardiometabolic risk factors (10). These findings might benefit the sporting industry by using dietary interventions in order to improve exercise performance and health recovery after exercise.

Recent experimental results have indicated that fructose containing drinks can increase exercise performance time, VO_2_ max, and TAS but decrease inflammatory markers and fatigue variables regardless of energy substrates. However, insulin response was slightly increased post consumption of blueberry sports drink and at recovery periods. This is because blueberry sports drink contains small amounts of organic sugar and blueberry fructose (11).

The purpose of this study was to determine the effect of blueberry supplementation on exercise performance time, inflammation markers, energy substrates, insulin, and TAS levels during two periods: non-supplemented period and supplemented period. We hypothesized that a blueberry supplement could serve as a countermeasure to oxidative stress, inflammation, fatigue materials, and energy substrate response, partially inhibit insulin responses, and increase exercise performance.

## Materials and Methods

### Subjects and Research Design

All subjects were recruited from exercise science major in some university in Busan, Korea. The sample size was eight healthy non-smoking student runners (male, n=8) at 23 yr of age who regularly participated in physical activity and other sports clubs. During the study, all subjects consented to exercise normally, maintain normal diet, weight status, and avoid the use of medications known to affect exercise performance time, inflammation markers, energy substrates, TAS, and insulin responses. Runners underwent two separateVO_2_ max tests (minimum of 1-week interval) divided into control period (1^st^) and blueberry supplemented (2^nd^) period.

All subjects signed informed consent forms and ethics were approved by the university.

### Blueberry supplementation and Exercise sessions

Blueberry was cultivated with fertile soil, lots of sunshine, organic soil, and a water reservoir. Blueberry fruits (140 g) were subjected to pulping after quick freezing at −80 °C for 30 min after cleaning. A small amount of distilled water was added after microwave assisted extraction. Supernatants were collected after centrifuging at 1200–1500 rpm for 90 sec. Aronia 15%, small amount of sugar, and refined water were then added.

VO_2_ max test was performed with a MMX3B portable gas analyzer (Germany) and ECG using Bruce protocol in DEU exercise science lab to evaluate the effect of blueberry supplementation on exercise performance after exercise on a treadmill. Expired respiratory gases were collected through open circuit spirometry during VO_2_ max test with MMX3B. To determine the VO_2_ peak, HR and rhythm were monitored continuously for arrhythmias and ischemia during the test via electrocardiography.

Following the test, subjects were also monitored for adverse symptoms until RPE, heart rate, VT, and blood pressure returned to normal levels as suggested by the ACSM to ensure subject safety before leaving the DEU exercise lab (11). All-out time was evaluated based on expected heart rate max, RPE, and respiratory ratio. RPE, VT, and AT RER were also evaluated with the MMX3B. Two separate experiments were performed for control period and blueberry supplemented period with a minimum interval of one week between the two periods.

### Blood Variables Analysis

Subjects came into the exercise physiology lab following 8–10 h fasting before pretest blood drawing. They did not perform exercise training or participate in specific sporting events within four days before taking the resting blood samples. Blood sample was taken by venipuncture at 30–40 min before the VO_2_ max test (11). During two separate experiments (interval of more than one week), blood was taken before exercise, the end of exercise, and 20 minutes of recovery time to compare the effect of blueberry supplementation. Each blood drawing collected 5 to 10 ml of blood. Blood was centrifuged to prepare serum and plasma. Samples were frozen at −80 °C for later analysis. Blood samples were assayed for markers of TAS, energy substrates, and IL-6 by assay kit and spectrophotometry. CRP level was analyzed with a Hitachi analyzer (Hitachi 7180, Japan) using immunoturbidometry and C-reactive protein reagent (Denka Co., Japan). Insulin level was evaluated with a gamma counter (1470 wizard, wallac, automatic counter, Finland) using coat-A-count kit (11).

### Statistical methods

All data are expressed as mean ± standard error. Data were compared between groups at each time point using Student’s *t*-test. Comparison between time points for each group was performed using independent *t*-test.

## Results

### Exercise Performance and Metabolic Variables following Blueberry Supplementation

Exercise performance time was significantly increased in the 2^nd^ period compare to that in the 1^st^ period. VO_2_ max was also significantly increased in the 2^nd^ periods compare to that in the 1^st^ period. However, there were no significantly differences in RPE, HRmax, or AT RER between the two periods (Table 1). VT was slightly faster in the 1^st^ period than that in the 2^nd^ period (data not shown).

**Table 1:** Exercise performance time and metabolic variables following blueberry intake at first and second trials

	***1st***	***2nd***	***P***
Time (sec)	763.00±43.15	812.50±43.38*	.041
RPE	18.87±1.06	17.22±0.86	.151
AT RER (CO_2_/O_2_)	1.18±0.34	1.11±0.56	.358
HRmax(beats/min)	194.27±4.57	191.61±7.21	.731
VO_2_ max(ml/kg/min)	45.36±6.15	51.24±4.82*	.048

RPE: ratings of perceived exertion

AT RER: anaerobic threshold RER

HRmax: heart rate max

### Blood Variables following Blueberry Supplementation

There were significant decreases of IL-6 and CRP levels during the recovery phase between the 1^st^ period and the 2^nd^ period. Glucose and insulin levels were significantly increased in the 2^nd^ period compare to those in the 1^st^ period in post session (end of exercise). TAS was also significantly increased in post and recovery sessions between the 1^st^ period and the 2^nd^ period. However, there were no significantly differences in TC, TG, HDL-C, LDL-C, FFA, or phosphorous levels between the two periods (Table 2).

**Table 2:** Blood variables following blueberry intake at first and second trials

		***Pre***	***Post***	***Recovery***
IL-6 (pg/mℓ)	1st	0.57±0.18	0.58±0.13	1.12±0.44
	2nd	0.56±0.15	0.56±0.11	0.62±0.17^*^
	*p*	.902	.902	.010
CRP (mg/dℓ)	1st	0.02±0.01	0.14±0.24	0.22±0.52
	2nd	0.02±0.02	0.03±0.03	0.02±0.01^*^
	*p*	.812	.205	.017
TC (mg/dℓ)	1st	165.38±21.37	177.25±20.37	150.37±16.07
	2nd	165.75±18.03	183.37±20.47	164.75±18.89
	*p*	.970	.558	.124
TG (mg/dℓ)	1st	85.25±22.02	113.75±48.79	97.75±38.00
	2nd	82.88±24.12	115.25±42.07	98.00±38.71
	*p*	.840	.948	.990
HDL-C (mg/dℓ)	1st	48.25±4.84	51.01±8.05	47.63±6.84
	2nd	50.35±5.52	56.82±8.09	53.30±7.38
	*p*	.432	.172	.134
LDL-C (mg/dℓ)	1st	95.33±15.84	106.87±19.43	89.37±12.05
	2nd	96.65±16.03	106.50±18.53	96.00±16.08
	*p*	.870	.969	.367
FFA (uEq/ℓ)	1st	350.88±147.09	474.12±206.14	329.50±105.43
	2nd	346.25±112.48	424.37±129.58	355.75±116.68
	*p*	.945	.573	.644
Phosphorus (mg/dℓ)	1st	4.21±0.48	4.95±0.67	3.85±0.50
	2nd	4.365±0.57	4.92±0.52	4.03±0.55
	*p*	.555	.926	.510
Glucose (mg/dℓ)	1st	81.00±5.63	99.25±9.73	93.87±12.83
	2nd	79.75±5.80	116.12±16.21^*^	91.75±14.49
	*p*	.669	.024	.761
Insulin (mIU/ℓ)	1st	5.34±1.67	5.27±1.09	10.53±6.46
	2nd	5.07±2.00	14.26±2.77^***^	16.19±7.57
	*p*	.777	.000	.130
TAS (mmol/ℓ)	1st	1.34±0.05	1.42±0.07	1.48±0.05
	2nd	1.31±0.05	1.50±0.08^*^	1.55±0.04^*^
	*p*	.316	.046	.018

CRP, c-reactive protein // FFA, free fatty acid // HDL-C, high density lipoprotein cholesterol IL-6, interleukin-6 // LDL-C, low density lipoprotein cholesterol // TAS, total antioxidant status TC, total cholesterol // TG, triglyceride //

* *P*<.05,

*** *P*<.001

## Discussion

Prolonged and intensive physical exercise can induce transient inflammation, oxidative stress, muscle damage, muscle soreness (10). It can also induce dehydration, sweating, and immune dys-function such as open window. Currently, ibuprofen is a popular drug used by athletes and runners to cope with physiologic demands of competition (10). However, it has safety and efficacy concerns. There is a growing interest in the use of fruit/vegetable extracts to reduce exercise induced physiologic dysfunction (11).

Some studies have followed athletes for a long period of time after strenuous physical exercise. Consuming polyphenol–rich supplements can decrease muscle damage, oxidative stress, immune dysfunction, and soreness with quicker recovery (5, 10, 12).

The aim of this study was to determine whether supplementation of blueberry could affect exercise performance time, VO_2_ max, inflammation markers, energy substrates, insulin level, and TAS level in pre-and post-intervention (1^st^ and 2^nd^ period). The main ingredients of blueberry are polyphenolic compounds, anthocyanins, myricetin, chlorogenic acid, arbutin, glycoside, and so on (13). Blueberries possess activities of neuroprotection, functional cognition (13), functional mobility (3), cardiotonic activity, anti-inflammatory effect, stress-induced skeletal muscle cell damage recovery (5), antioxidants (2), anti-tumor, immune response, and allergy responses, especially in a Turkish population (14).

This study revealed that exercise performance time was significantly increased in the 2^nd^ period compare to that in the 1^st^ period. VO_2_ max was also significantly increased in the 2^nd^ period. However, there were no significantly differences in RPE, HRmax, or AT RER between the 1^st^ period and the 2^nd^ period.

Healthy students consuming blueberry exhibited enhanced exercise performance time and VO_2_ max. Blueberry polysaccharides might have increased the ability of aerobic metabolism and exercise endurance irrespective of fatigue variables or CRP levels. Our results are consistent with those of previous studies on animals (15). We have evaluated the effect of fructose supplementation on cardiopulmonary function, lactate levels, inflammatory markers, and exercise performance in healthy students (11) and found that fructose supplementation can increase exercise performance time and VO_2_ max but decrease CRP and lactate levels (11).

Results of the present study are consistent with results of the New Zealand group study in terms of fatigue recovery (9). There were significant decreases of IL-6 and CRP during recovery between the 1^st^ period and the 2^nd^ period. Glucose and insulin levels were also significantly increased in post session (end of exercise) between the two periods. TAS was also significantly increased in post and recovery sessions between the 1^st^ period and the 2^nd^ period. However, there were no significantly differences in TC, TG, HDL-C, LDLC, FFA, or phosphorous levels between the 1^st^ period and the 2^nd^ period. Our results are also, consistent with many previous studies showing that supplementation of blueberry can increase antioxidant levels but decrease inflammation levels (10, 11, 16). Subjects fed blueberry might be better protected against oxidative stress after exhaustive exercise compared to subjects fed a control diet as reported previously (8, 11). Such results also might be caused or changed by polyphenol in the blueberry supplementation (11).

In this study, we found that fructose supplementation increased antioxidant levels but decreased inflammatory markers. However, phosphorous level was not significantly changed while insulin level was increased. These findings may benefit the sporting fields and community who could consider ergogenic intervention to specifically target health and exercise performance. In fact, insulin levels were not significantly changed by the supplementation of fructose than those by glucose. This study showed that insulin levels were increased which is caused by small amounts of sugar in the blueberry.

Fructose is commonly utilized in the sports fields because it is not likely to induce insulin responses. Therefore, it is widely used as an immediate energy source, especially in the sports fields. In addition, blueberry contains polyphenols with antiviral effect against cellular pathogens.

Further studies are necessary to confirm these findings and determine whether blueberry supplementation can provide soluble factors that exert immune responses, especially in athletes. Future research will also determine whether longer-term blueberry intake in athletic diet can reduce physiologic stress of heavy exertion, increase recovery speed, and produce other benefits such as neuronal signaling in the brain, functional mobility, mediating memory function, neurodegenerative disease, and dementia.

## Conclusion

The blueberry supplementation can potentially increase the exercise performance, metabolic variables and decrease the IL-6 and CRP levels caused by an increased TAS level.

## Ethical considerations

Ethical issues (Including plagiarism, informed consent, misconduct, data fabrication and/or falsification, double publication and/or submission, redundancy, etc.) have been completely observed by the authors.
